# Patients’ experiences of cold exposure during ambulance care

**DOI:** 10.1186/1757-7241-21-44

**Published:** 2013-06-06

**Authors:** Jonas Aléx, Stig Karlsson, Britt-Inger Saveman

**Affiliations:** 1 Department of Nursing, Umea University, SE-901 87, Umea, Sweden

**Keywords:** Cold exposure, Comfort zone, Finger temperature, Thermal comfort, Thermal discomfort, Patients’ experience

## Abstract

**Background:**

Exposure to cold temperatures is often a neglected problem in prehospital care. Cold exposure increase thermal discomfort and, if untreated causes disturbances of vital body functions until ultimately reaching hypothermia. It may also impair cognitive function, increase pain and contribute to fear and an overall sense of dissatisfaction. The aim of this study was to investigate injured and ill patients’ experiences of cold exposure and to identify related factors.

**Method:**

During January to March 2011, 62 consecutively selected patients were observed when they were cared for by ambulance nursing staff in prehospital care in the north of Sweden. The field study was based on observations, questions about thermal discomfort and temperature measurements (mattress air and patients’ finger temperature). Based on the observation protocol the participants were divided into two groups, one group that stated it was cold in the patient compartment in the ambulance and another group that did not. Continuous variables were analyzed with independent sample t-test, paired sample t-test and dichotomous variables with cross tabulation.

**Results:**

In the ambulance 85% of the patients had a finger temperature below comfort zone and 44% experienced the ambient temperature in the patient compartment in the ambulance to be cold. There was a significant decrease in finger temperature from the first measurement indoor compared to measurement in the ambulance. The mattress temperature at the ambulance ranged from −22.3°C to 8.4°C.

**Conclusion:**

Cold exposure in winter time is common in prehospital care. Sick and injured patients immediately react to cold exposure with decreasing finger temperature and experience of discomfort from cold. Keeping the patient in the comfort zone is of great importance. Further studies are needed to increase knowledge which can be a base for implications in prehospital care for patients who probably already suffer for other reasons.

## Background

It is estimated that approximately 280 million people live in the circumpolar regions [1]. Circumpolar environmental conditions like those found in Sweden are characterized by considerable fluctuations in temperature—long, cold, and dark winters and short summers [2].

Thermal comfort is defined in the present study as, “a subjective response, or state of mind, where a person expresses satisfaction with the thermal environment” ([3], s.21). People experience a wide range of temperatures between cold and heat. Within this range, skin temperatures over about 43°C and below about 15°C trigger not only a sensation of thermal discomfort, but also feelings of pain [4-6]. When the body is in the comfort zone, skin temperature varies from 31 – 34°C [7]. A change in skin temperature greater than ±2°C is considered large enough to elicit statements of thermal discomfort [8]. Several studies have clearly concluded that women have a lower hand temperature than men even at thermo-neutrality [4,9]; in cold conditions, women will therefore experience discomfort earlier than men [10,11].

Cold exposure increases discomfort and, if untreated, causes incremental disturbances of vital body functions until ultimately reaching hypothermia [12]. The initial reaction to cold exposure is peripheral vasoconstriction leading to peripheral blood being shunted to central body regions in order to support vital organs and to retain body heat [13,14]. Tonus of skeletal muscle increases (“shivering”), which can effectively increase heat generation up to four times [13,15].

Hypothermic patients (having a core body temperature <36°C) [16] are affected with a higher range of physiological and pathological responses and dysfunctions [14], like increased oxygen consumption [17], inhibition of drug metabolism [18,19], disturbance of blood clotting mechanisms [20-22], changes in mental status [23], and a longer duration of hospital stay [24], compared to normothermic patients.

Cold exposure may also impair cognitive function, increase pain, aggravate thermal discomfort, and contribute to fear and an overall sense of dissatisfaction [25,26]. Onset of rain and wind significantly increases the thermal and physical effects of the environment on already affected people [27].

A study by Henriksson et al. [28] has shown that insulation and protection from cold are important in prehospital care and also that the polyester blankets used in ambulance care are essentially worthless in windy conditions. In a study by Aléx et al. [29] patients injured outdoors described how they struggled, trying to move from side to side, to avoid contact with the ground and that protecting themselves from the chill that came from the ground was very important and necessary. It was described as if cold was creeping through their bodies from the ground, causing them to experience thermal discomfort from the cold.

Caring for comfort is the goal of helping patients reach well-being [30]. In ambulance care, it is important for nursing staff to identify and prevent heat loss to avoid patients’ thermal discomfort and hypothermia. Still, exposure to cold temperatures is often a neglected problem in prehospital care [12]. Hypothermia does not occur only for patients lying outside but can also be found in non-traumatized indoor patients [31]. Ambulance care especially with respect to patients ‘experience of thermal discomfort is rarely described in the research literature on prehospital care. In terms of cold exposure, ambulance care may be particularly challenging in circumpolar regions with conditions like those in Sweden.

## Aim

The aim of this study is to investigate injured and ill patients’ experiences of cold exposures and to identify related factors during ambulance care.

## Method

### Design

The present study is a field study in ambulance care performed in northern Sweden.

### Procedure

**Figure 1:**
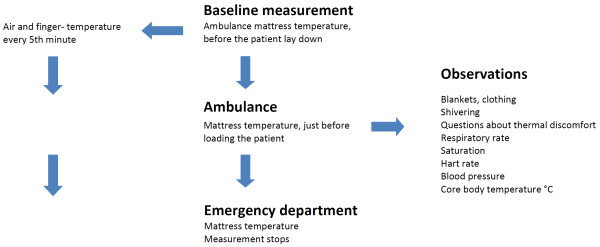
Observation schedule.

The field study was based on observations and measurements (see Figure 1). The first author (JA) accompanied one ambulance for 22 days during the day from January to March 2011. The observations ranged from 5 minutes to 45 minutes (mean = 20 ± 8.25), depending on the length of time of the ambulance transport. The consecutively selected patients were observed when they were cared for by ambulance staff in prehospital care in Umeå in the north of Sweden. Mean outdoor temperature during observation days was −8.6°C, ranging from +7°C to −25°C. Patients who were included in the study were ≥18 years old and spoke Swedish. Unconscious patients or those having difficulty communicating were excluded from the study. The patients included in the study were provided information about the study and asked to participate. The baseline observations and temperature measurement began after the ambulance staff examined the patients’ vital parameters, assessed their medical history. For the observation procedures, measurements of O_2_, heart rate, blood pressure, (Lifepak 12, USA) respiratory frequency, and core body temperature (internal ear, measured with Braun ThermoScan , Exac Temp IRT 4520, Germany) were collected by the ambulance staff. Finger temperature (on outer fingertips, left hand) and the mattress temperature were measured with an infrared (IR light, which is invisible to the human eye) thermometer (CIR 8819) with dual laser points, (indicating the measurement area) while the air temperature was measured with an extern sensor (Bead probe 6030) both by the researcher. In the ambulance, the patient was asked questions such as, Do you feel cold? Do you experience discomfort from cold? Do you experience the mattress to be cold? These were answered with “yes” or “no”. Observations were made of things like patient clothing and shivering. At the emergency department (ED) or other acute medical care facility the patient was being taken to, the observations and measurements stopped.

### Sample

The study included 62 patients, 34 women and 28 men. Ages ranged from 21 to 98 years old (mean = 67.0 ± 22.9, see Table 1). A description of the conditions prior to the ambulance transport based on data from the Emergency Service Center is presented in Table 2.

**Table 1:** Comparison of patient data between the group who reported cold (n = 27), and those who did not (n = 35)

	**Total**	**Experience of cold**	**No experience of cold**	**p-value**	**phi**
**Age**					
≤ 59	21	10	11	*0.512*	
≥ 60	41	17	24		
**Sex**					
Male	28	13	15	*0.875*	
Female	34	14	20		
**Clothing**					
Thin	21	11	10		
Medium	31	12	19		
Thick	10	4	6		
**Observation start**					
Indoor	54	22	32		
Outdoor	8	5	3		
**Observation time (minutes)**		18	21		
**Cold mattress**					
Yes	14	12	2	*0.001*	0.46
No	48	15	33		
**Shivering**					
Yes	15	9	6	*0.018*	0.34
No	47	5	42		
**Thermal discomfort from cold**					
Yes	14	14	0	*0.001*	0.62
No	48	35	13		

**Table 2:** Conditions described by the Emergency Service Center prior to ambulance transport (n = 62)

	
Cardiac diseases	11
Respiratory insufficiency	8
Minor trauma	8
Internal medicine, various	8
Hip fractures	6
Abdominal pain	5
Orthopedic, various	4
Psychiatric /intoxication	4
Seizure	2
Bleeding, large	1
Unclear	5

### Analysis

Based on the observation protocol, participants were initially divided into two groups, one group that reported the experience of that it was cold in the patient compartment in the ambulance and another group that did not report that it was cold in the patient compartment in the ambulance. Next, comparisons were made between those who reported the mattress to be cold with those who did not. Data collected from the observation protocol and data from measurements of O_2_ levels, respiratory frequency, heart rate, and blood pressure were statistically analyzed. Continuous variables were analyzed with independent sample t-test, paired sample t-test, and dichotomous variables with cross tabulation (Chi square test and Fisher’s exact test; significance level 0.05). To quantify the size of the difference between groups, standardized effect size (ES) was calculated, along with Cohen ’s d for parametric data and Phi for non-parametric data. All statistical analyses were performed with SPSS software (version 18.0, SPSS Inc., Chicago, IL, USA).

### Ethics

The study was approved by the Regional Ethics Committee, Umeå, Sweden (Dnr. 2010-391-31 M). The participants were provided information about the study and informed that their participation was voluntary and that they had the right to withdraw at any time without affecting their care.

## Results

Of the 62 participating patients, 42 (67%) had a finger temperature below the comfort zone of 31°C at baseline. In the ambulance, 52 patients (85%) had a finger temperature below the comfort zone and 27 patients (44%) reported the experience of the ambient temperature in the patient compartment in the ambulance to be cold. At baseline, surface temperature on the ambulance mattress ranged between −11°C and 24.3°C (mean = 14.3°C) and at the ambulance temperature ranged from −22.3°C to 8.4°C (mean = −3.9°C; Table 3).

**Table 3:** Mattress, air and patient finger temperature at baseline, in the ambulance and at the emergency department (n = 62)

	**Baseline**			**Ambulance**			**Emergency department**		
	**Min**	**Max**	**Mean**	**Min**	**Max**	**Mean**	**Min**	**Max**	**Mean**
**Mattress**	−11.0^3^	24.3^3^	14.3^3^	−22.3^1^	8.4^1^	−3.9^1^	−3.3^4^	22.1^4^	14.1^4^
**Air**	−22.9^3^	25.5^3^	17.2^3^	5.2^1^	27.1^1^	18.7^1^	12.3^2^	25.3^2^	19.9^2^
**Finger**	11.4	37.8	27.6	12.0	36.3	25.0	15.4	37.8	26.1

^1^ 3 missing, ^2^ 6 missing, ^3^ 1 missing, ^4^ 2 missing.

When the ambulance staff arrived, 21 of the patients were wearing thin clothes, like t-skirts, and nightdresses, with one patient wearing no clothes at all. Almost all patients (n = 59) were exposed to an air temperature <24°C in the ambulance. Internal ear temperature ranged from 35.5°C to 39.5°C (mean 37.1°C, ± 0.9).

A significant difference was found between the patients that reported cold and those who did not concerning the mattress (p = 0.001; ES = 0.46), shivering (p < 0.018; ES = 0.34), and thermal discomfort from cold (p < 0.001; ES = 0.62) (see Table 1).

Among the 54 indoor patients, there was a significant decrease in finger temperature from baseline measurement indoors (mean = 28.4 ± 4.47) to measurement in the ambulance (mean = 25.2, ± 5.08; Cl 2.15 to 4.15; p = 0.001; ES = 0.66).

Between the group that reported the mattress to be cold in the ambulance, and the other group that did not, there was also a significantly lower mattress temperature for those who reported the mattress to be cold (p = 0.036; ES = 0.77). Finger temperature showed a small difference (ES = 0.35), and the air temperature showed no significant difference between groups (see Table 4).

**Table 4:** Comparison of mattress, air and patient finger temperature between the groups who reported the mattress to be cold (n = 14) and those who did not (n = 48)

	**Experience of cold matt**		**No experience of cold matt**		**p-value**	**Cohen’s d**
	**Mean**	**SD**	**Mean**	**SD**		
**Mattress**	−8.45	9.16	−2.52^1^	5.97^1^	0.036	0.77
**Air**	18.73	3.79	18.70^1^	4.62^1^	0.982	0.01
**Finger**	26.15	5.56	28.06	5.51	0.270	0.35

^1^ 3 missing.

No significant difference was found between women and men with the respect to the report of cold ambient temperature (p = 0.789) or shivering (p = 0.557). There were no significant differences between the two groups in terms of core body temperature, including vital parameters, among those who experienced the mattress to be cold and those who did not. The measurements of O_2_ levels, respiratory frequency, heart rate and blood pressure showed no significant differences between groups.

## Discussion

The present study reveals that there is a problem of cold ambient temperature in ambulance care and also that patients have negative experiences and physiological reactions to cold exposure denote to decreased finger temperatures. From the results in this study, it is confirmed that the ambient temperature in the patient compartment is too low to be comfortable. More research is needed to confirm that this is true for other circumpolar regions as well. However, this may also be a problem for indoor patients lying on the floor for several hours [32] and in subtropical climates where trauma patients are at risk of developing hypothermia [33].

Out of 62 patients, nearly half thought it was cold in the patient compartment in the ambulance and a majority (59 patients) were exposed to an air temperature <24°C in the patient compartment in the ambulance. Elderly patients suffering from hypo-metabolic diseases may feel discomfort from cold even in air temperature of 24°C [32]. The very old, children, and the chronically ill are at higher risk of hypothermia in cold-susceptible environments [34]. In a study of thermal comfort, preferred room temperature for patients was 23.9°C in winter and 24.6°C in summertime [35]. The patients in this study were exposed to cold ambient temperatures (c.f. Table 3), increasing thermal discomfort.

One fourth of the patient’s reported that the mattress was cold when lying on the stretcher in the ambulance and Phi indicated a medium effect size (0.46) between the experience of cold and the experience of a cold mattress. Body heat is lost to the environment by convection, such as with wind, radiation, and evaporation, and through conduction, when the body is in direct contact with cold surfaces [28,34]. Conduction is an effective method of heat transfer [21]. Cooling of the back and chest are the leading influences of the overall sensation of discomfort from cold [36]. If an injured or ill person is lying on a cold surface, heat loss by conduction increases significantly [13,28]. Early application of adequate insulation, in addition to immediate care for life-threatening conditions, to reduce heat loss and prevent core body temperature cooling is an important intervention in prehospital care [37]. A study by Lundgren and Henriksson [38] shows that warming modalities are effective for patients and prevent further decrease of core body temperature. In this study, the patients were embedded in a polyester synthetic blanket to protect their body integrity but also to protect them from cold. It is well known that contact with metal increases heat loss but surprisingly the material on the stretcher, plastic and polyester synthetic blankets, adapted to the ambient temperature almost immediately when the patients were outside and when staff prepared for transport. The ambulance mattress appears to have been an important factor in cooling the patient. In the present study, it seems that patients were warming up the stretcher, and not the other way around.

The present study shows that those who reported the patient compartment to be cold also shivered (ES = 0.34). Shivering however can be precipitated for reasons other than from cold exposure, e.g. as a result of drugs [39]. For each 1°C fall in core body temperature, the musculature maximal power falls by 3% and mechanical efficiency is simultaneously reduced [40]. Shivering can increase oxygen demand up to 500% which might be detrimental for patients with cardiac dysfunction [41]. At minor trauma events outside in wintertime, shivering has been described as the worst experience from the injury because of the lack of bodily control and as devastating for those suffering from, for example, fractures. Supplying active heat decreased shivering regardless of whether the shivering developed from fear or pain [29]. The experience of cold is subjective and thermal discomfort can vary with the conditions people are expected to be in. It is possible that people do not report thermal discomfort even though the environment may be out of the comfort zone, if they do not expect a better condition [42]. Following our results and those of other researchers, it is important to provide prehospital patients a warm patient compartment in the ambulance and active heat supply to minimize discomfort from cold as well as shivering.

In the present study, there was a significant decrease in finger temperature between measurements of temperature indoors compared to inside the ambulance compartment (ES = 0.66). People can only be in a comfort zone when heat production and heat loss are balanced [43]. Candas and Dufour [10] clearly state that there is a relationship between skin temperature and discomfort. Even in normothermic patients, the cold stress response renders thermal discomfort, which increases the experience of anxiety and pain [44]. The experience of thermal sensation and thermal discomfort are controllers of thermoregulation irrespective of changes in temperature [45]. A study of whole-body cold discomfort [46] showed that when finger temperature was above 30°C, there was no experience of cold discomfort. But when finger temperature was below 30°C, cold discomfort was a possibility. The present study has shown that, at baseline, only one third of the patients were in the skin temperature comfort zone and in the ambulance only 15% had a finger temperature in the comfort zone, i.e. patients had a finger temperature that was below the comfort zone when the ambulance staff arrived, which then decreased even further during prehospital care. The results in the present study indicate the importance of the possibility of providing heat to patients to increase the skin temperature into the comfort zone.

Patients’ core body temperature in the present study was on average 37°C. This high mean temperature may be dependent on the fact that one fifth of the patients had a fever, which contributed to a normothermic mean temperature. The experience of cold is subjective and we considered including patients with fever as they also can report being cold. In our study, the change in core body temperature between groups was not significant, which is not surprising because the cold exposure from the ambient temperature was apparently too short. A longer cold exposure probably would have resulted in a decreased core body temperature.

In the present study, women did not report their discomfort from cold higher than men. These results differ from other studies finding that women react earlier to cold stress then men [4,9,10]. Further studies are needed.

### Methodological considerations

In this study no significant differences between the sexes were found. This may be explained by the sample size or length of time in the ambulance. The time for the observations, i.e. wintertime, was chosen to be sure of an exposure of cold temperatures. All data was collected by the first author using the same measurement instruments for all observations. Two observations were done to test the observation protocol before the study started. The relevance of the protocol was checked and found valid, which strengthens the present study. The results are clinically significant even if there were few participants. Another limitation is that patients’ medical condition, e.g. metabolic diseases and medication such as vasoconstrictors were not assessed. These factors may contribute to the test results. It is also well known that patients in pain are influenced by sympathetic nervous reactions, which also lead to vasoconstriction. The patients participating in this study are probably comparable to typical patients in prehospital care with respect to age, proportion of men and women, and reasons for ordering an ambulance.

## Conclusion

In wintertime, the climate is cold in the north of Sweden and cold exposure is common in prehospital care. Sick and injured patients immediately react to cold exposure with decreasing finger temperature and reporting experience of discomfort from cold. Keeping the patient in the comfort zone is of great importance. Further studies are needed to increase knowledge that can be a base for implications in prehospital care for patients who probably already suffer for other reasons.

## Competing interests

The authors declare that they have no competing interests.

## Authors’ contributions

JA: Planning the study, data collection, analysis and writing of the manuscript. SK: Analysis and supervising of the manuscript. B-IS: Supervising and planning the study, analysis and writing of the manuscript. All authors have participated to the manuscript according to the criteria for authors. All authors read and approved the final manuscript.

## Authors’ information

Jonas Aléx is an RN, a PEN and a PhD student. Sig Karlsson is an RN, PhD and professor, Britt-Inger Saveman is an RNT, PhD, FEANS and professor. All are employed at the Department of Nursing, Umeå University. B-I Saveman is also the pro-Director at the National Centre of Disaster Medicine, Umeå University, Sweden.
